# Increased risk for the development of postoperative severe hypoxemia in obese women with acute type a aortic dissection

**DOI:** 10.1186/s13019-019-0888-9

**Published:** 2019-04-25

**Authors:** Ming Gong, Zining Wu, Shijun Xu, Lei Li, Xiaolong Wang, Xinliang Guan, Hongjia Zhang

**Affiliations:** Department of Cardiac Surgery, Beijing Aortic Disease Center, Beijing Anzhen Hospital, Capital Medical University, Beijing Institute of Heart Lung and Blood Vessel Diseases, Beijing Laboratory for Cardiovascular Precision Medicine, and Beijing Engineering Research Center of Vascular Prostheses, No.2 Anzhen Street, Beijing, 100029 China

**Keywords:** Body mass index, Cardiovascular disease, Gender, Hypoxemia, Risk factors

## Abstract

**Background:**

The purpose of this study is to identify the risk factors for postoperative severe hypoxemia after surgery for acute type A aortic dissection.

**Methods:**

This was a single-center retrospective study including 112 consecutive patients undergoing urgent aortic arch surgery for acute type A aortic dissection between December 2016 and April 2017 at Beijing Anzhen Hospital.

**Results:**

Multivariate logistic regression analysis identified female (OR, 12.978; 95% CI, 3.332 to 50.546; *p* < 0.001) and increased body mass index (OR, 1.473; 95% CI, 1.213 to 1.789; *p* < 0.001) as independent predictors of postoperative severe hypoxemia in patients with acute type A aortic dissection.

**Conclusions:**

Obesity and female were independent risk factors for postoperative severe hypoxemia in patients with acute type A aortic dissection. More attention should be paid to preventing postoperative severe hypoxemia in obese women with acute type A aortic dissection.

## Background

Due to its high rates of mortality and morbidity, acute type A aortic dissection is one of the most urgent surgical intervention [1]. Despite improved perioperative management and surgical techniques, the patients who undergo cardiac surgery generally have an increased risk of postoperative hypoxemia. Following cardiopulmonary bypass (CPB), postoperative hypoxemia has been reported to occur in 12.2–27.1% of patients, and after aortic dissection surgery, this figure is as high as 51% [2, 3]. As a dangerous complication, hypoxemia is usually accompanied by several unfavourable consequences, including prolonged ventilator support, increased hospital length of stay, increased intensive care unit (ICU) length of stay, and higher perioperative mortality. However, data pertaining to the incidence and risk factors of severe postoperative hypoxemia are scarce for patients with acute type A aortic dissection. Therefore, it is essential to investigate the risk factors associated with severe postoperative hypoxemia after surgery for acute type A aortic dissection. Research in this area, by facilitating early intervention and treatment for hypoxemia, is expected to improve the surgical treatment effect and reduce perioperative mortality rates.

## Methods

### Patient population

From December 2016 and April 2017, electronic medical records and laboratory results were reviewed for a total of 112 patients with acute Stanford type A aortic dissection. All patients underwent urgent aortic arch surgery with CPB, involving moderate hypothermic circulatory arrest (HCA) at Beijing Anzhen Hospital. All aortic arch replacements, with or without aortic valve operations, were eligible. Patients who died intraoperatively or within 24 h postoperatively were also excluded since no meaningful data were available for the evaluation of severe postoperative hypoxemia.

### Study design

In this single-centre retrospective study, the analytical focal points included the preoperative characteristics, operative details, and postoperative outcomes of 112 consecutive patients (83 men and 29 women; age range, 24–74 years; average age, 47.7 ± 10.7 years). Each patient suffered from acute Stanford type A aortic dissection and underwent aortic surgery at Beijing Anzhen Hospital. The hospital’s Ethics Committee approved this study protocol. Acute type A aortic dissection was diagnosed using enhanced computed tomography (CT) scanning, while aortic valve regurgitation was confirmed using echocardiography. All procedures were performed by the same surgery team.

The Berlin definition [4] proposes 3 categories of hypoxemia based on the degree of the condition: first, mild (200 mmHg < PaO2/FIO2 ≤ 300 mmHg); second, moderate (100 mmHg < PaO2/FIO2 ≤ 200 mmHg); and third, severe (PaO2/FIO2 ≤ 100 mmHg). In the present study, according to the diagnostic criteria for acute respiratory distress syndrome (ARDS) established by the Berlin definition, severe postoperative hypoxemia was defined as a PaO2/FiO2 ≤ 100 mmHg. In our study, all patients breathed spontaneously with nasal prongs or face masks with inhaled oxygen for 5–8 L/min prior to surgical intervention. The arterial blood gas and arterial partial pressure of oxygen to fraction inspired oxygen (PaO2/FiO2) were calculated for the perioperative period. Noteworthily, most patients with severe postoperative hypoxemia were identified within 72 h of receiving surgery, and we evaluated the condition within 72 h after each patient’s arrival at the ICU. Therefore, 112 patients were divided into 2 groups according to postoperative PaO2/FiO2: firstly, a non-severe hypoxemia group (*n* = 71); and secondly, a severe hypoxemia group (*n* = 41). In the event that more than one result was available for a given variable, the worst daily value was collected on days 0–3 of the perioperative period. Body mass index (BMI) values were recorded on admission using each patient’s height and weight [BMI = weight (kg) / height (m)^2^]. The primary endpoint of this study was to evaluate the incidence and risk factors of severe postoperative hypoxemia in 112 patients with acute type A aortic dissection.

### Surgical procedures

**Fig. 1:**
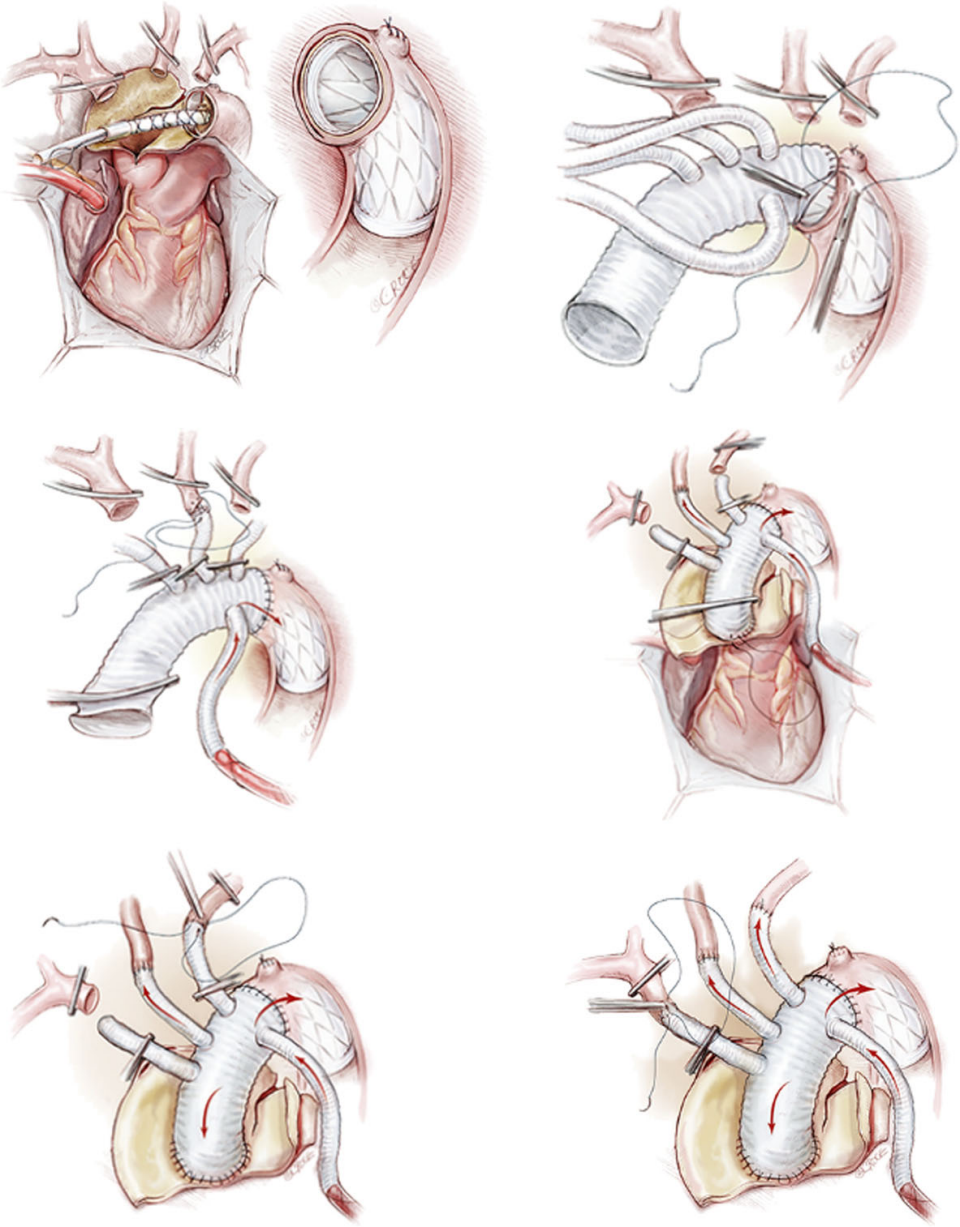
The total arch replacement using a tetra-furcate vascular graft in combination with the implantation of a special stented graft into the descending aorta

Standard anaesthetic management was used with endotracheal intubation. The procedure refers to total arch replacement using a tetra-furcate vascular graft in combination with the implantation of a special stented graft into the descending aorta (Fig. 1). Briefly, the procedure is performed with right axillary artery cannulation for CPB and antegrade cerebral perfusion [5–15 mL / (kg·min)] under moderate HCA. After systemic heparinisation (300 U / kg body weight and maintaining an activated clotting time longer than 480 s), CPB was established. During CPB, temperature-adjusted flow rates of 2.5 L / (min·m^2^) were used, and the mean arterial pressure was generally maintained between 50 and 70 mmHg. Our policy was to excise completely the primary tear based on the extent of the disruption in each case. This procedure involves the implantation of a stented graft into the descending aorta, total arch replacement with a 4-branched vascular graft, and a specific sequence for aortic reconstruction (proximal descending aorta, then the left carotid artery, ascending aorta, left subclavian artery, and finally innominate artery). After completing distal anastomosis, CPB was reinstituted, and after a 5-min period of cold reperfusion for free radical washout, the patient was gradually rewarmed to a normal temperature. Proximal anastomosis was then performed.

### Statistical analysis

The normality of the data distribution was tested using the Kolmogorov-Smirnov test. Data are expressed as the mean ± standard deviation (SD) for continuous data with a normal distribution, as the median (25th percentile and 75th percentile) for continuous data with a non-normal distribution, or counts and percentages for categorical values. For comparison, one-way analysis or the Wilcoxon rank sum test was used for continuous variables, while the chi-square test or Fisher’s exact test was used for categorical variables. Logistic regression models were used to identify univariate and multivariate predictors for severe postoperative hypoxemia. Univariate logistic regression analysis was used first to identify possible risk factors for severe postoperative hypoxemia, and the multivariate model included variables that were found significant in the univariate analysis. In addition, to evaluate the effects of BMI and gender on postoperative levels of PaO2/FiO2 over time, we created a mixed-effect analysis of variance model. For all analyses, SPSS 18.0 (SPSS, Inc., Chicago, IL) was used, and a probability value of less than 0.05 was considered statistically significant.

## Results

### Baseline characteristics

After applying the exclusion criteria, the total study cohort consisted of 112 consecutive patients with a mean age of 47.7 ± 10.7 years (range 24–74 years). Of these patients, 83 were male and 29 were female. Most of the patients with acute type A aortic dissection had chest pain (94.8%) as the predominant preoperative symptom. As shown in Table 1, which provided an overview of patient characteristics and perioperative variables, the incidence of severe postoperative hypoxemia in our patient population was 36.6% (41/112). In order to gain insight into the factors associated with severe postoperative hypoxemia, the patient population was divided into 2 groups based on each individual’s postoperative PaO2/FiO2: firstly, patients with postoperative PaO2/FiO2 ≤ 100 mmHg were classified into the severe hypoxemia group; and secondly, the other patients were classified into the non-severe hypoxemia group.

**Table 1:** Perioperative characteristics of patients with acute type A aortic dissection

Characteristics	Non-severe hypoxemia (*n* = 71)	Severe hypoxemia (*n* = 41)	*p* value
Demographic data			
Age, year	46.6 ± 11.1	49.5 ± 9.8	0.17
Female	13 (18.3)	16 (39.0)	0.02
BMI, kg/m^2^	24.9 ± 3.3	28.5 ± 3.9	< 0.001
Onset to surgery, hour	48 (24, 120)	30 (21, 72)	0.19
Medical history			
Hypertension	55 (77.5)	34 (82.9)	0.49
Diabetes mellitus	1 (1.4)	5 (12.2)	0.05
Bicuspid aortic valve	4 (5.6)	0	0.31
Cerebrovascular disease	3 (4.2)	3 (7.3)	0.79
Coronary artery disease	2 (2.8)	3 (7.3)	0.53
Smoking history	34 (47.9)	21 (51.2)	0.74
Marfan syndrome	3 (4.2)	4 (9.8)	0.45
COPD	1 (1.4)	2 (4.9)	0.63
Preoperative condition			
Lactate, mmol/L	1.6 ± 0.7	1.9 ± 1.2	0.09
PaO2/FiO2, mmHg	276 ± 87	249 ± 77	0.10
D-dimer, ug/mL	1.1 (0.6, 2.8)	2.3 (0.8, 3.1)	0.05
White blood cell, × 10^3^ cells/uL	10.3 ± 3.5	12.8 ± 3.6	< 0.001
Hemoglobin, g/dL	137 ± 18	134 ± 17	0.28
sCr, umol/L	83.2 ± 28.8	92.0 ± 29.9	0.13
Troponin I, ng/mL	0.02 (0.00, 0.06)	0.02 (0.00, 0.08)	0.96
Aortic root size, mm	41.1 ± 8.9	39.4 ± 4.9	0.21
Severe aortic regurgitation	16 (22.5)	8 (19.5)	0.87
Ascend aorta size, mm	45.8 ± 7.5	44.5 ± 6.7	0.36
Left ventricular ejection fraction, %	62.5 ± 6.2	63.8 ± 5.9	0.31
Hemopericardium	6 (8.5)	5 (12.2)	0.76
Operation details			
The duration of operation, hour	8.0 ± 1.8	9.0 ± 1.7	0.004
CPB time, min	200 ± 51	232 ± 59	0.004
Aortic cross clamp time, min	120 ± 46	132 ± 35	0.14
The duration of HCA, min	27.2 ± 9.8	28.0 ± 6.9	0.64
Nasopharyngeal temperature, °C	23.2 ± 1.9	22.4 ± 1.5	0.02
Rectal temperature, °C	25.5 ± 2.3	25.2 ± 2.3	0.47
Intraoperative blood loss, mL	1449 ± 657	1578 ± 811	0.36
Intraoperative amount of plasma, mL	400 (0, 400)	500 (100, 800)	0.004
Intraoperative amount of PRBCs, mL	300 (0, 600)	600 (150, 750)	0.13
Postoperative outcomes			
Length of ICU, day	1.7 (1.0, 3.0)	6.0 (3.5, 11.0)	< 0.001
In-hospital mortality	1 (1.4)	5 (12.2)	0.05
Reoperation for bleeding	2 (2.8)	7 (17.1)	0.02
Postoperative dialysis	6 (8.5)	17 (41.5)	< 0.001
Low cardiac output syndrome	2 (2.8)	3 (7.3)	0.53
Sepsis	9 (12.7)	8 (19.5)	0.33
Paraplegia	2 (2.8)	2 (4.9)	0.97
Cerebral infarction or bleeding	0	7 (17.1)	0.001

Values are mean ± SD, n (%), or median (interquartile range)

*BMI* body mass index, *COPD* chronic obstructive pulmonary disease, *CPB* cardiopulmonary bypass, *HCA* hypothermic circulatory arrest, *ICU* intensive care unit, *PaO2/FiO2* arterial partial pressure of oxygen to fraction of inspired oxygen, *PRBCs* packed red blood cells, *sCr* serum creatinine

Among the preoperative characteristics, Table 1 showed that BMI values were higher for the severe hypoxemia group when compared to the non-severe hypoxemia group (*p* < 0.001). Compared to the non-severe hypoxemia group, the total proportion of severe hypoxemia was greater for women than for men in severe hypoxemia group (39.0% vs. 18.3%; *p* = 0.02). Notably, no significant differences in medical history were observed between 2 groups. Hypertension was present in 89 of the 112 patients, and with regard to aortic pathology, 6.3% of patients suffered from Marfan syndrome. On admission, clotted false lumen was found on enhanced CT in 56 patients. 89 patients received dissections that extended below the diaphragm, while for 23, the dissection terminated above the diaphragm. There were no significant differences between 2 groups with respect to standard laboratory tests and preoperative imaging tests, except for white blood cell counts (*p* < 0.001).

### Operation details

Operation details are presented in Table 1. Not surprisingly, although no significant differences were observed in the duration of aortic cross-clamp and HCA, patients with severe hypoxemia required longer operations and CPB time when compared to patients with non-severe hypoxemia (*p* = 0.004 and *p* = 0.004). Moreover, compared to their counterparts with non-severe hypoxemia, patients with severe hypoxemia had greater levels of intraoperative plasma (*p* = 0.004). Nasopharyngeal temperature values were also significantly different between the groups (*p* = 0.02).

### Postoperative outcomes

Postoperative clinical details are summarised in Table 1. Overall in-hospital mortality was 5.4% (6/112) for patients with acute type A aortic dissection. The causes of death were acute heart failure (*n* = 2) and multi-organ failure (n = 2), sepsis (*n* = 1), and respiratory failure (*n* = 1). In the patient population, in-hospital mortality was 12.2% (*n* = 5) for patients suffering from severe hypoxemia, while it was 1.4% (*n* = 1) for patients non-severe hypoxemia patients. Among the postoperative characteristics, postoperative clinical outcomes were complicated in patients with severe hypoxemia, reflecting a high rate of complications. As expected, patients with severe hypoxemia had a longer ICU length of stay when compared to their non-severe counterparts (*p* < 0.001).

The incidence of severe postoperative hypoxemia is summarised in Table 2. In the patient population, women presented more frequently for severe postoperative hypoxemia compared to men (55.2% vs. 30.1%). Furthermore, a directly proportional relationship was observed between BMI and the incidence of severe postoperative hypoxemia (13.3, 46.2 and 73.3%, *p* < 0.001).

**Table 2:** Incidence of postoperative severe hypoxemia by BMI and gender categories in patients with acute type A aortic dissection

		BMI		
		Normal: < 25	Overweight: 25 to 30	Obesity: > 30
Gender	Female	17	10	2
Severe hypoxemia		5 (29.4)	9 (90.0)	2 (100.0)
Non-severe hypoxemia		12 (70.6)	1 (10.0)	0
Gender	Male	28	42	13
Severe hypoxemia		1 (3.6)	15 (35.7)	9 (69.2)
Non-severe hypoxemia		27 (96.4)	27 (64.3)	4 (30.8)

Values are n (%)

*BMI* body mass index

The chi-square test for trend is significant for the association between incidence of hypoxemia and increasing BMI categories and gender (*p* < 0.001)

### Multivariate logistic regression analysis

**Fig. 2:**
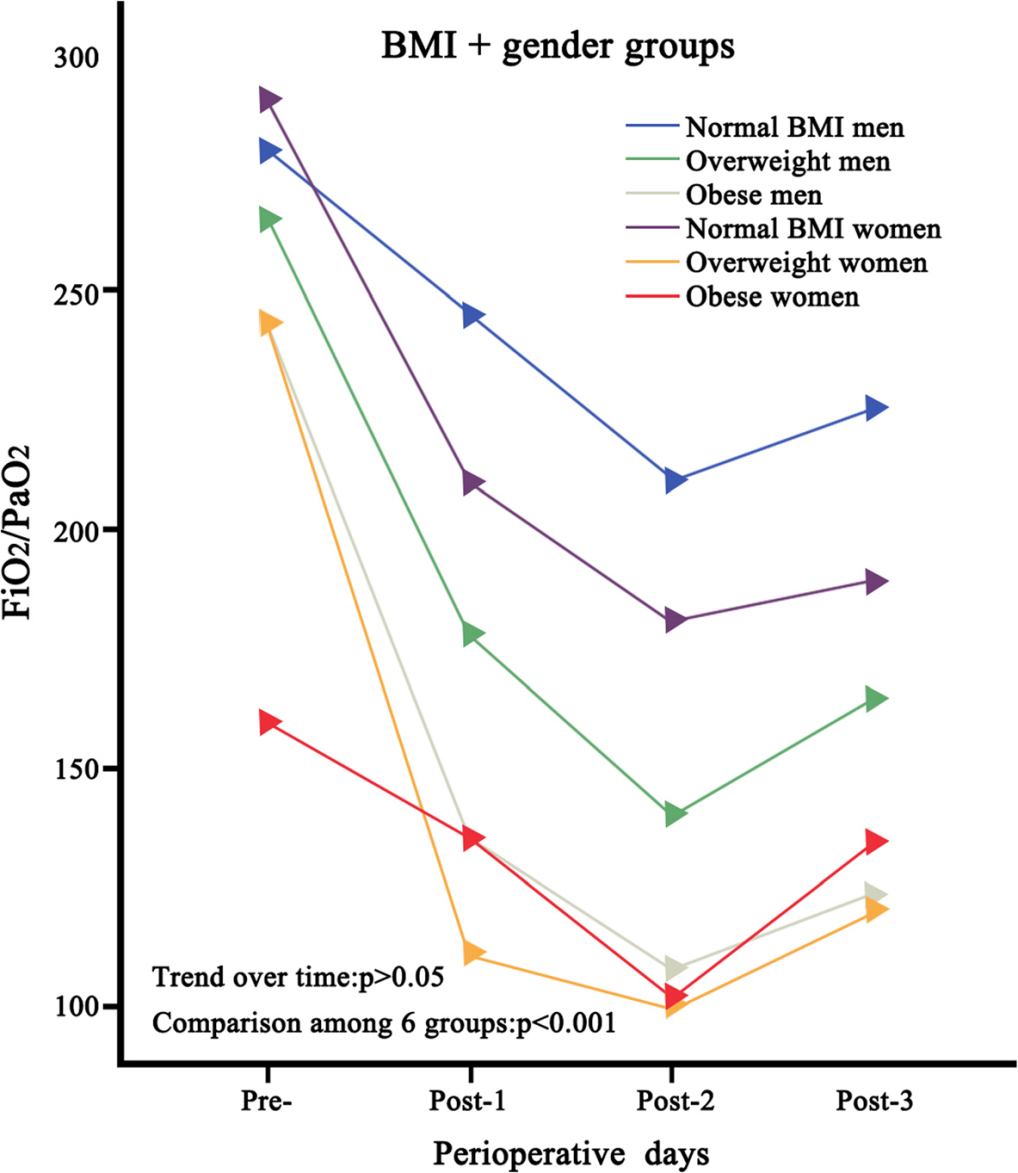
Changes in PaO_2_/FiO_2_ among BMI + gender groups over time during perioperative period

In a primary model, all preoperative risk factors and intraoperative parameters of recognised clinical significance were included. Significant differences were found between non-severe hypoxemia patients and severe hypoxemia patients in terms of gender, BMI, preoperative white blood cell counts, duration of operation, CPB time, nasopharyngeal temperature, and intraoperative plasma levels. In addition, mixed-effect analysis of variance modelling was undertaken to evaluate the impact of BMI and gender on severe postoperative hypoxemia (Fig. 2). Figure 2 showed that female patients with increased BMI values were associated with a higher incidence of severe postoperative hypoxemia compared to male patients with lower BMI values (*p* < 0.001). Moreover, these significantly different trends did not change with postoperative time (*p* > 0.05).

Table 3 provides an overview of the risk factors for severe postoperative hypoxemia identified using multivariate logistic regression. From the analysis, gender [odds ratio (OR), 12.978; 95% confidence interval (CI), 3.332 to 50.546; *p* < 0.001] and BMI [OR, 1.473; 95% CI, 1.213 to 1.789; *p* < 0.001] were identified as independent risk factors for severe postoperative hypoxemia. Similarly, multivariate logistic regression analysis confirmed that obese female patients were independently associated with severe postoperative hypoxemia in acute type A aortic dissection (OR, 2.591; 95% CI, 1.664 to 4.035; *p* < 0.001).

**Table 3:** Risk factors for postoperative severe hypoxemia in multivariate logistic regression analysis in patients with acute type A aortic dissection

Clinical variables	OR	95% CI	*p* Value
Female	12.978	3.332 to 50.546	< 0.001
BMI, kg/m^2^	1.473	1.213 to 1.789	< 0.001
Female + BMI	2.591	1.664 to 4.035	< 0.001
Diabetes mellitus	7.111	0.673 to 75.184	0.10
White blood cell, ×10^3^ cells/uL	1.158	0.988 to 1.358	0.07
The duration of operation, hour	1.150	0.794 to 1.667	0.50
CPB time, min	1.008	0.995 to 1.021	0.21
Nasopharyngeal temperature, °C	0.776	0.537 to 1.121	0.18

*BMI* body mass index, *CPB* cardiopulmonary bypass, *CI* confidence interval, *OR* odds ratio

## Discussion

Ever since deep hypothermia and selective cerebral perfusion techniques were introduced into surgical interventions for acute Stanford type A aortic dissection, the incidence of postoperative cerebral complications has decreased. Postoperative hypoxemia, as another life-threatening postoperative complication for acute Stanford type A aortic dissection, may be attributed to prolonged mechanical ventilation and ICU length of stay. It is a common complication after coronary artery bypass grafting (CABG) or valve replacement [5–7]. However, few studies [3] have been conducted on postoperative hypoxemia for acute type A aortic dissection. Given that mild or moderate hypoxemia was excessively common in patients with acute type A aortic dissection, and since hypoxemia patients usually did not present with cardiogenic pulmonary oedema or typical radiological pulmonary infiltrates, a close link has been discerned between severe postoperative hypoxemia and prolonged ICU length of stay, prolonged mechanical ventilation, and higher hospitalisation costs. Therefore, for the purpose of improving clinical outcomes, it is particularly important for us to conduct an intensive study addressing the risk factors for severe postoperative hypoxemia after surgery for acute type A aortic dissection.

Compared to other elective cardiac surgical procedures, acute type A aortic dissection surgery is associated with a high incidence of postoperative hypoxemia [5–7]. In this study, the incidence of severe postoperative hypoxemia was identified as 36.6% (41/112) for acute Stanford type A dissection surgery. Acute type A aortic dissection indicates a sudden rupture of the intima, propagation of the dissection into the medial layer, acute bleeding, and activation of a systemic inflammatory response. The mechanism of hypoxemia after surgery for acute aortic dissection remains unclear. In our opinion, imbalance of ventilation and perfusion leads to hypoxemia primarily in the process of acute bleeding. Alongside this, inflammatory cascade reaction significantly increases pulmonary vascular pressure and results in impaired alveolar surfactant function [3, 8]. As this complication has an adverse effect on a patient’s postoperative trajectory, timely treatment is critical. Therefore, practitioners must try their best to maintain the perioperative fluid balance and reduce the systemic inflammatory response, especially several days after surgery.

Previously reported risk factors for hypoxemia after cardiac surgery included chronic obstructive pulmonary disease, advanced age, obesity, history of smoking, haemodynamic instability, complex cardiac surgery, previous heart surgery, emergency surgery, preoperative myocardial infarction, preoperative diabetes, pulmonary oedema, postoperative lung infections, excessive blood transfusion, and prolonged CPB time [6–9]. If we could determine the predictors of severe postoperative hypoxemia, then it would be a more feasible prospect to treat hypoxemia in a timely way. In the present study, we identified obesity and gender as the risk factors for severe postoperative hypoxemia after acute Stanford type A aortic dissection surgery.

The prevalence of obesity in Western countries is increasing. In the USA, one-third of the population is obese, and two-thirds are overweight [10]. It is well-known that obesity is a multifactorial disorder which is frequently accompanied by serious comorbidities and complications, including the risk of severe cardiovascular diseases and respiratory diseases [11, 12]. Moreover, research indicates that weight loss is associated with a marked improvement in pulmonary function [13]. Obesity has also been reported as a predictor of hypoxemia [11, 14]. Consistently, we found that obese patients are more likely to develop severe postoperative hypoxemia. In the present study, BMI showed a significant influence for severe postoperative hypoxemia in both the univariate analysis and multivariate regression models.

Decrease in lung compliance is particularly obvious among obese patients. Therefore, breathing difficulties clearly increase for individuals who suffer from obesity. In addition, respiratory resistance has been shown to be increased among the obese. Studies have shown that the occurrence of ARDS is related to an imbalance of anti-inflammatory and pro-inflammatory cytokines, as well as oxidants and anti-oxidants [15–18]. Most obese patients suffer from chronic and excessive inflammation and oxidative stress [15, 16]. Abnormal cytokine products and acute phase reactants are significantly greater for obese patients, while pro-inflammatory signalling pathways are markedly upregulated. Furthermore, induction of pro-inflammatory cytokines and mediators has been shown to increase as a consequence of weight gain [17, 18]. Moreover, obesity can increase oxidative stress and reactive oxygen products, which may result in direct damage to the cellular membranes, monocytes cellular adhesion, and the release of chemotactic factors and vasoactive substances. At the same time, the high burden of comorbidities and underlying renal damage in obese patients represents another way to account for higher levels of severe postoperative hypoxemia. Therefore, this study’s results emphasise that more attention must be paid to the prevention of severe postoperative hypoxemia among obese patients.

The female gender is generally considered a risk factor for cardiac surgery. In particular, the impact of female gender on clinical outcomes after isolated CABG has been well reported in multiple studies. In their meta-analysis, Alam et al. [19] demonstrated that women who underwent isolated CABG experienced higher mortality at follow-up compared to men. In the risk models of both the Society of Thoracic Surgeons and the European System for Cardiac Operative Risk Evaluation, the female gender was also listed as one of the risk factors for cardiac surgery [20, 21]. Although gender-related differences in patients undergoing CABG have been well investigated, relatively few reports have been published regarding such differences in patients undergoing acute type A aortic dissection. Nevertheless, given the important public health implications of research in this area, gender-related differences in patients receiving this surgery are worth exploring. Fukui et al. [22] found that although there was a tendency towards a statistically significant difference based on Kaplan–Meier analysis, multivariate analysis revealed that the female gender was not an independent predictor of late mortality. Moreover, data from the International Registry of Acute Aortic Dissection (IRAD) [23] showed that aortic dissection occurred in women on average 6 or 7 years later than in men. Thus, it is worth emphasising that gender differences in patients undergoing surgical repair of acute type A aortic dissection have been a matter of debate.

In the present study, multivariate analysis demonstrated that gender has a significant impact on the rate of severe postoperative hypoxemia after surgical repair for acute type A aortic dissection. Since IRAD was a multicentre study, the surgical teams involved and the methods used were not uniform. By contrast, activities in the present study were performed in a single centre and the surgical method was uniform. In addition, the results indicated that female patients also tend to suffer from a greater number of preoperative comorbidities, including diabetes and anaemia. Although the prevalence of these conditions was not observed to be significantly different between the genders in this research, it is easy to surmise that these critical conditions may affect postoperative outcomes. Therefore, these findings are expected to promote adequate treatment for female patients undergoing acute type A aortic dissection.

### Study limitations

As a retrospective studies, the present study has several potential limitations. First, the patient population was relatively small and only associated with a single institution, which makes it subject to inherent selection and information biases. Second, the influence of factors such as the experience of the individual surgeon and institutional philosophy on the decision made regarding the treatment decision was not taken into account for this analysis. Third, considering the emergency nature of Stanford type A aortic dissection surgery, the identified risk factors may have been confounded by the complex interactions among different organ systems.

## Conclusions

In conclusion, our study demonstrated that two independent risk factors for severe postoperative hypoxemia in patients undergoing acute type A aortic dissection were BMI (specifically, obesity) and gender (specifically, female). Therefore, appropriate preventive measures ought to be taken to minimise the incidence of severe postoperative hypoxemia in obese women with acute type A aortic dissection. These measures may include maintaining the perioperative fluid balance and reducing the systemic inflammatory response.

## Abbreviations

ARDS: Acute respiratory distress syndrome
BMI: Body mass index
CABG: Coronary artery bypass grafting
CI: Confidence interval
CPB: Cardiopulmonary bypass
CT: Computed tomography
HCA: Hypothermic circulatory arrest
ICU: Intensive care unit
IRAD: International Registry of Acute Aortic Dissection
OR: Odds ratio
PaO2/FiO2: Arterial partial pressure of oxygen to fraction of inspired oxygen
SD: Standard deviation
